# Supplementary material for: Nationwide genome surveillance of carbapenem-resistant Pseudomonas aeruginosa in Japan
Source: Antimicrob Agents Chemother. 2024 Apr 2;68(5):e01669-23. doi: 10.1128/aac.01669-23 (PMC11064530; doi:10.1128/aac.01669-23)
Supplement: Fig. S1 — Secondary structure alignment of IntA (JBP_33480) and IntB (JBP_33500) of JBBCAEG-19-0032. [file aac.01669-23-s0001.docx]

Conservation: 9

IntA_JBBCAEG-19-0032 1 MARNH----------LRSKTG-QGR---YES--------------KARRVLIPEFRLLR-INFLE----- 36

IntB_JBBCAEG-19-0032 1 MSIIAH---------LQFSQVQDHQLPDRCNSAS-------QYLN-VDDNFVISRTAQG-VIGSIYGDDE 52

Int_lambda_NP_040609.1 1 MGR-------------------------RRS--------------HERRDLPPNLYIRN-NGYY------ 24

IntI1_R388_HXF36_RS00185 1 MKTAT----------------------------------------------------------------- 5

IntB_6945_BBM67682.1 1 MYSIS--HDHA----LFKK---------YALPI-------DEAYPMPTARTVVSVKRDG-SPASYFEDDA 47

IntA_6945_BBM67680.1 1 MEHFYH---------LVAT---------------------SRSIEI----GDIELGDPP-GSSDFT---- 31

IntB_6283_BCN27153.1 1 MIKISDLEHQNKL--LKAQQN-IGAHTNYLIDVLKPAIERGDWDELE--ALDIGFTVTGESLGKIADDEA 65

XerD_Eco_IEU92_RS14660 1 MKQ--DLARIEQ---------------------------------------------------------- 10

Int_SXT_AAL59748.1 1 MAL-SVSW-------LDARLN-KEA--------------KETVVKADRDGLSARVSPK------------ 35

IntA_6283_BCN27155.1 1 MIGTHEKQSFDIYDDYWLLSIEVDTLAKLQNYA---------NIDI--DTGEVTHSPYLSP--------- 50

[Consensus_aa:](http://prodata.swmed.edu/promals3d/info/consensus.html) **M**....................................................p*h*...............

[Consensus_ss:](http://prodata.swmed.edu/promals3d/info/consensus_ss.html) hhh eeeee

Conservation: 5

IntA_JBBCAEG-19-0032 37 -----THNDGNPKGLPDYE----------------------------FSSAEDSYLPNFPVLIQGH---- 69

IntB_JBBCAEG-19-0032 53 WNVKM--YDAAGICVYNFSAWTEGITTPLVTVIAGEMKKIQIARMYLFQKPRKPNS----LRLGD----- 111

Int_lambda_NP_040609.1 25 ----------------------------------------------CYRDPRTGKE----FGLGRD---- 40

IntI1_R388_HXF36_RS00185 ----------------------------------------------------------------------

IntB_6945_BBM67682.1 48 WDFND-LFNTKNEKKSNYT----------------------------LTF--QAQKHN------------ 74

IntA_6945_BBM67680.1 32 ------VSKPMHFDGVNLL----------------------------FTS--NGE--------------- 50

IntB_6283_BCN27153.1 66 WRNLQSHIEPQPKKFYTLE----------------------------FTN--DGRVVE------------ 93

XerD_Eco_IEU92_RS14660 ----------------------------------------------------------------------

Int_SXT_AAL59748.1 36 -----------GKIVFQFR----------------------------YRF--DGKQQR--VDIGTYPLMK 62

IntA_6283_BCN27155.1 51 ------SNLKSRLKKTDII----------------------------IAP--DGT--------------- 69

[Consensus_aa:](http://prodata.swmed.edu/promals3d/info/consensus.html) ...............................................*h*p...ptp...............

[Consensus_ss:](http://prodata.swmed.edu/promals3d/info/consensus_ss.html) ee e

Conservation:

IntA_JBBCAEG-19-0032 70 ----------------------------------------------GEPWAIGNLYLTTKLQREAG---- 89

IntB_JBBCAEG-19-0032 112 LNKLARLALNNNLPLSDLFDDANLNRLLLPS-----FAE-LDRNPMRGILQLLKELFDIRVKHPDFK--- 172

Int_lambda_NP_040609.1 41 RRIAITEAI-----------QANIELFSGHKH-KPLTAR-INSDNSVTLHSWLDRYEKILASRG------ 91

IntI1_R388_HXF36_RS00185 6 -----------------------------------------APLPPLRSVKVLDQLRERIRYLH------ 28

IntB_6945_BBM67682.1 75 PKLLLEFKQ----------------------------------------------RMYWLIWGGKDTLLS 98

IntA_6945_BBM67680.1 51 --PEFYANA----------------------------------------------FIMSRRIVE------ 66

IntB_6283_BCN27153.1 94 RNLKNELKV---------------------------------------------LILKMMWLSPHD---- 114

XerD_Eco_IEU92_RS14660 11 -------------------------------------------------------FLDALWLEKN----- 20

Int_SXT_AAL59748.1 63 LAEARNELD-----------RLRAVLDQGRNPKLYLQQERAKYSANQSFESIFRDWIDSAGKQG------ 115

IntA_6283_BCN27155.1 70 ------------------------------------------------IVYPQSLYLVSKLRGE------ 85

[Consensus_aa:](http://prodata.swmed.edu/promals3d/info/consensus.html) .......................................................*hh*....b........

[Consensus_ss:](http://prodata.swmed.edu/promals3d/info/consensus_ss.html) hhhhhhhhh hhhhhhhhhhhhh

Conservation: 7 5

IntA_JBBCAEG-19-0032 90 ---------YESRTFRG-IADHLLDY-LRFLEDEGMD---YMHF---------------PKNDRLKVTYR 130

IntB_JBBCAEG-19-0032 173 -------FAPSDYTIIECMQALYNKHPKRQQHDPQQT---------------------------KLIPSR 208

Int_lambda_NP_040609.1 92 ---------IKQKTLIN-YMSKIKAI-RRGLPDAPLE---------------------------DITTKE 123

IntI1_R388_HXF36_RS00185 29 ---------YSLRTEQA-YVNWVRAF-IRFHGVRHPA---------------------------TLGSSE 60

IntB_6945_BBM67682.1 99 MEGKTFRKIEQVKDIQV-HVDLLLRV-FANTANNAFS---------------------------YISNDI 139

IntA_6945_BBM67680.1 67 ----------GVKDTEP-TSYALLRF-FRYLGKNNLN---------------------------WND--- 94

IntB_6283_BCN27153.1 115 ---------HSFCTLYE-ALNDLKKV-INPLLNEGINTLS------------------------ALDFDQ 149

XerD_Eco_IEU92_RS14660 21 ---------LAENTLNA-YRRDLSMM-VEWLHHRGLT-------------------------LATAQSDD 54

Int_SXT_AAL59748.1 116 ---------LKEKTWHY-QKRSSEIYLLPRLG--KYP-------------------------LTDINELS 148

IntA_6283_BCN27155.1 86 ---------AAVKDTGS-IAKGLLAF-TRYLDSTHYS---QVDEDGYEIPPEYLTYKTLSKYEEEGVPWR 141

[Consensus_aa:](http://prodata.swmed.edu/promals3d/info/consensus.html) .............s*h*...*h*...*h*..*h*....*h*.p..*h*s.............................s..p

[Consensus_ss:](http://prodata.swmed.edu/promals3d/info/consensus_ss.html) hhhhhh hhhhhhhh hhhhhh h hhh

Conservation: 5

IntA_JBBCAEG-19-0032 131 YRQRLI---------------------------------------------------------------- 136

IntB_JBBCAEG-19-0032 209 LYAALI---------------------------------------------------------------- 214

Int_lambda_NP_040609.1 124 IAAMLN---------------------------------------------------------------- 129

IntI1_R388_HXF36_RS00185 61 VEAFLS---------------------------------------------------------------- 66

IntB_6945_BBM67682.1 140 VFSQIKESLRGWSEKSCATRLNALSVLVQVNLHFPERARFHIPYQEGETARKVSKKYAAHGKGHFPTVIP 209

IntA_6945_BBM67680.1 95 ----ENEEL----ERYP----------------------------------------------------I 104

IntB_6283_BCN27153.1 150 LETWVLTDFTDI-DFEREKIYNGLNRLYLEARGLP----FKVNLNKKLTASDFGLTLKEAK---QYTVIP 211

XerD_Eco_IEU92_RS14660 55 LQALL----------------------------------------------------------------- 59

Int_SXT_AAL59748.1 149 LRNCL----------------------------------------------------------------- 153

IntA_6283_BCN27155.1 142 FAEFLL---------------------------------------------------------------- 147

[Consensus_aa:](http://prodata.swmed.edu/promals3d/info/consensus.html) *h*...*l*.................................................................

[Consensus_ss:](http://prodata.swmed.edu/promals3d/info/consensus_ss.html) hhhhhh

Conservation:

IntA_JBBCAEG-19-0032 137 ---------------------------------DFVTAGLL--------------------SASTASARI 153

IntB_JBBCAEG-19-0032 215 ---------------------------------AALNL-----------------------ELDDFNAIA 228

Int_lambda_NP_040609.1 130 ---------------------------------GYIDEG----------------------KAASAKLIR 144

IntI1_R388_HXF36_RS00185 67 ---------------------------------WLANERKV--------------------SVSTHRQAL 83

IntB_6945_BBM67682.1 210 VI------------------YEQYL-------SRLIQDVESAYRDFLGGRDLESEVEAEYRSLQTEHDIE 254

IntA_6945_BBM67680.1 105 YL------------------FRNYL-------DKQIAKGNM--------------------RRSVGASTL 129

IntB_6283_BCN27153.1 212 QRLYYMGLQKSEEVINEAYALRHELEQLADYITTYFEK----------AYVGY--------AKYLVSGDA 263

XerD_Eco_IEU92_RS14660 60 --------------------------------AERLEGGY---------------------KATSSARLL 76

Int_SXT_AAL59748.1 154 --------------------------------REVSES-----------------------SPSNTERLV 168

IntA_6283_BCN27155.1 148 ---------------------------------ANCKNIEGS-----NGDEAL--------SLSTAKSYM 171

[Consensus_aa:](http://prodata.swmed.edu/promals3d/info/consensus.html) ...................................*h*p........................p..s.p...

[Consensus_ss:](http://prodata.swmed.edu/promals3d/info/consensus_ss.html) hhhhhh hhhhhhhh

Conservation: 6

IntA_JBBCAEG-19-0032 154 NAVVNFYRRIINWN------------------------------LVS-----DANIEGVAFTEIKKYISV 188

IntB_JBBCAEG-19-0032 229 ESIAELYKQKNENT------------------------------LFG-----IPECRSRWYKNPIVWGDA 263

Int_lambda_NP_040609.1 145 STLSDAFREAIAEG------------------------------HIT----------------------- 161

IntI1_R388_HXF36_RS00185 84 AALLFFYGKVLCTD------------------------------LPW----------------------- 100

IntB_6945_BBM67682.1 255 QEIETLYQELIVDI------------------------------EVEEKAKNSG-------------IFP 281

IntA_6945_BBM67680.1 130 SVIRRFYVFCYRHG------------------------------YVE----------------------- 146

IntB_6283_BCN27153.1 264 RQKNGGITWYLVKSGERNKKRTTAFQQAFTALRSPNEEEVLALAQLHKPQIKSE---------------- 317

XerD_Eco_IEU92_RS14660 77 SAVRRLFQYLYREK------------------------------FRE----------------------- 93

Int_SXT_AAL59748.1 169 SVLHKFYDWLIDEQ------------------------------ILE----------------------- 185

IntA_6283_BCN27155.1 172 SAVIGFYKWMQKYG------------------------------YIP-----NNDQNVVTHYTTRTRHYE 206

[Consensus_aa:](http://prodata.swmed.edu/promals3d/info/consensus.html) p.*l*..*h@*pb*h*b.................................b*h*........................

[Consensus_ss:](http://prodata.swmed.edu/promals3d/info/consensus_ss.html) hhhhhhhhhhhh

Conservation: 5

IntA_JBBCAEG-19-0032 189 TSNYGIPR------------------------------NLMVNSHNLAIANTKKQSQAEYINDGGDL-RP 227

IntB_JBBCAEG-19-0032 264 IQQHGLAS------------------------------AFESRS-------------------------I 278

Int_lambda_NP_040609.1 162 -TNHVAATR-----------------------------AAKSEVRR----------------------SR 179

IntI1_R388_HXF36_RS00185 101 ----LQEIG-----------------------------RPRPSR---------------------RLPVV 116

IntB_6945_BBM67682.1 282 QEVLDDYRDKAKDEVESIYRSKAHRGVIDAYKKDNVLLDVKAY---------------------AQL-KG 329

IntA_6945_BBM67680.1 147 -KLPFEVHGH----NKYGQML------TDITIK-----SPKQE---------------------TEL-TP 178

IntB_6283_BCN27153.1 318 ---YIDKFH-----------------------------SERQLT-------------------IGQW-TI 335

XerD_Eco_IEU92_RS14660 94 -DDPSAHLA-----------------------------SPKLPQ--------------------RLP-KD 112

Int_SXT_AAL59748.1 186 -INAAAGIT-----------------------------AKKVSG-----------------KKGKRT-RV 207

IntA_6283_BCN27155.1 207 LNHHDM-LAHTKPGSEHSNET------SNIMRM-----FPKKDT------------TPA----YKKL-KP 247

[Consensus_aa:](http://prodata.swmed.edu/promals3d/info/consensus.html) .p....................................s.c........................p*h*.p.

[Consensus_ss:](http://prodata.swmed.edu/promals3d/info/consensus_ss.html)

Conservation: 59 9 6

IntA_JBBCAEG-19-0032 228 LTVSEQSYMLQALLES---SREYQLMFYFALFTGARVQTIGTLRIKHLLG-GLDSN------GYLRLPIG 287

IntB_JBBCAEG-19-0032 279 ENWIDLKAYLGELQ--------TAAKYWIHFFTGMRSNEARHLPADSYTT-IKVNG------ADVHILRG 333

Int_lambda_NP_040609.1 180 LTADEYLKIYQAAESS---PCWLRLAMELAVVTGQRVGDLCEMKWSDIV------D------GYLYVEQS 234

IntI1_R388_HXF36_RS00185 117 LTPDEVVRILGFLE------GEHRLFAQLLYGTGMRISEGLQLRVKDLD----FDH------GTIIVREG 170

IntB_6945_BBM67682.1 330 VTEKQAIDAFRMI--------EGACFGACSAFTGMRISELTQIEGDSYKEVD-IDG------VKLCTMRS 384

IntA_6945_BBM67680.1 179 MNDLDIRHIRDNWHRAG-ISGEFRLLVATVINSGLRAIEVADIKPRHFK----VPKGFKG--KTYTDIKI 241

IntB_6283_BCN27153.1 336 TNRTEAQSLFKMLN--------GGCLWGLMARTGMRGDEMYSLNTVQGCTKDIIDR------QNIYVIHA 391

XerD_Eco_IEU92_RS14660 113 LSEAQVERLLQAPLIDQPLELRDKAMLEVLYATGLRVSELVGLTMSDIS----LRQ-------GVVRVIG 171

Int_SXT_AAL59748.1 208 LNDNEIRILWRYLHESK-ITEKNRIYIKLLLLLGGRKGELIQAEKHHFD----LQS-------AMWTVPI 265

IntA_6283_BCN27155.1 248 MHPEHKAIFNEHATSL---PKAANLIFRLCLEAGLRIDEATHFPSSDIG----IADCSDLDVVPFKITRT 310

[Consensus_aa:](http://prodata.swmed.edu/promals3d/info/consensus.html) *h*s..c...*h*.p.*h*...........*hhh*.*hhh*.*h***G**.**R**..**E***h*.p*h*..pp*h*.....*h*..........*h*.*h*...

[Consensus_ss:](http://prodata.swmed.edu/promals3d/info/consensus_ss.html) hhhhhhhhhhhh hhhhhhhhhhhhh hhhhh hhh eeeeeee

Conservation: 9

IntA_JBBCAEG-19-0032 288 -ASTLVDTK-----NGKR---MTLLVPG---WVVQDMLVYCRSAEAKKRRARSYYG-------------- 331

IntB_JBBCAEG-19-0032 334 -----YTSKIAGQ-NHTE---TFWMTAG---IIEKGIAAALHIGKIAALINNFDD--------------- 376

Int_lambda_NP_040609.1 235 --------K-----TGVK---IAIPTAL----HIDALGISMK-----ETLDKCKE--------------- 264

IntI1_R388_HXF36_RS00185 171 --------K-----GSKD---RALMLPE---SLAPSLREQLSRARAWWLKDQAEGRSGVALPDALERKYP 221

IntB_6945_BBM67682.1 385 --WTDKLDK-----LSRE---DAWACAPICEKALLILTVLNDKYRSVSG--------------------- 423

IntA_6945_BBM67680.1 242 GPSHGCYTK-----YSKN---RTISMPV---WLMEQVTQYCESERYKERKRLYFFN-------------- 286

IntB_6283_BCN27153.1 392 -----NLSKTVKGSQSIQDEFVTTEIGM---KAYEVLQALHTPLRKRD---------------------- 431

XerD_Eco_IEU92_RS14660 172 --------K-----GNKE---RLVPLGE---EAVYWLETYLEHGRPWLL--------------------- 201

Int_SXT_AAL59748.1 266 -----EIRK-----QGEK---IGAPIMR---PLIKPAIELIELAMQM----------------------- 296

IntA_6283_BCN27155.1 311 --------K-----GSKP---RIVEVPI---TLYEELEIYKESHARLKKLNKRNDLIQ------------ 349

[Consensus_aa:](http://prodata.swmed.edu/promals3d/info/consensus.html) ........**K**......t.p....*hh*.*h*s.....*hh*b.*h*..*h*.c............................

[Consensus_ss:](http://prodata.swmed.edu/promals3d/info/consensus_ss.html) e eeee h hhhhhhhhhhhhhhhhhhhhhh

Conservation: 6

IntA_JBBCAEG-19-0032 332 ----DTEDN--------------YVF-------------------LSKN-----------GVPYYTSKKE 353

IntB_JBBCAEG-19-0032 377 ----SDPSQY-------------PLFPSLVKPRKKEIRVFAGGPVISQS-----------GRSVS----- 413

Int_lambda_NP_040609.1 265 ----ILGGE--------------TII-------------------ASTR-----------REPLS----- 281

IntI1_R388_HXF36_RS00185 222 RAGHSWPWF--------------WVF-------------------AQHTHSTDPRSGVVRRHHMY----- 253

IntB_6945_BBM67682.1 424 ---DIHLSPRFSLKGEGKGWTGDTIH-------------------RQLK-----------DAQLH----- 455

IntA_6945_BBM67680.1 287 ---TGDEDA--------------PVF-------------------ITKE-----------GNRFKEE--- 306

IntB_6283_BCN27153.1 432 -----SSSQ--------------SFF-------------------HKTTEDFS----KVGKASIG----- 454

XerD_Eco_IEU92_RS14660 202 ---NGVSID--------------VLF-------------------PSQR-----------AQQMT----- 219

Int_SXT_AAL59748.1 297 -----SKST--------------YLF-------------------PANG-----------QEELA----- 312

IntA_6283_BCN27155.1 350 SGKETDTIE--------------YLF-------------------LSNK-----------GKPYS----- 370

[Consensus_aa:](http://prodata.swmed.edu/promals3d/info/consensus.html) ........................*l@*....................ppp..............*h*......

[Consensus_ss:](http://prodata.swmed.edu/promals3d/info/consensus_ss.html) ee e

Conservation:

IntA_JBBCAEG-19-0032 354 ILDRRDSRISVGAGMADRASEVSIQDGAALRTHI-------QQILLPRIH-------------------- 396

IntB_JBBCAEG-19-0032 414 --------------------------QQRLLARWPDLIIQEADIRELEQ-------------FDGFRDWR 444

Int_lambda_NP_040609.1 282 --------------------------SGTVSRYF-------MRARKA----------------------- 295

IntI1_R388_HXF36_RS00185 254 --------------------------DQTFQRAF-------KRAVEQ----------------------- 267

IntB_6945_BBM67682.1 456 --------------------------TKNLRQIF-------YDYSLHIDIRYLPEEMDEIFNLLNPIVHE 492

IntA_6945_BBM67680.1 307 -----------------------GQRSNSIDTLW-------GRLRNAIK------------ENS------ 328

IntB_6283_BCN27153.1 455 --------------------------KYSMAWFK-------NALREELAL--TNEDLTDL-KVSDPNLS- 487

XerD_Eco_IEU92_RS14660 220 --------------------------RQTFWHRI-------KHYAVLAGI-------------------- 236

Int_SXT_AAL59748.1 313 --------------------------TNGFDTTI-------PNNVKIWAR------RSL----------- 332

IntA_6283_BCN27155.1 371 --------------------------VNTLETDF-------SKLRRRIRE-------------------- 387

[Consensus_aa:](http://prodata.swmed.edu/promals3d/info/consensus.html) ............................s*h*...*h*........p*h*..........................

[Consensus_ss:](http://prodata.swmed.edu/promals3d/info/consensus_ss.html) hhhhhhhh hhhhhhh

Conservation: 9 69 5 5 5 69 6 9

IntA_JBBCAEG-19-0032 397 --------RGNPEFQ---YFTFHDLRATFGMNLLESQLQHMGDQSITAALEYVQQRMGHSNKETTMQYLS 455

IntB_JBBCAEG-19-0032 445 NDPEV----TAGVVW---PLGTHQCRRSLAVYCARSGL---------VSVGSLGLQFKHLTEVMASYYRK 498

Int_lambda_NP_040609.1 296 ----------SGLSFEGDPPTFHELR-SLSARLYEKQ----------ISDKFAQHLLGHKSDTMASQYRD 344

IntI1_R388_HXF36_RS00185 268 ----------AGITK---PATPHTLRHSFATALLRSG----------YDIRTVQDLLGHSDVSTTMIYTH 314

IntB_6945_BBM67682.1 493 KLSPIKENERGELYW---RFNTHSLRRTFAHFVVGHGL---------VSLASLKHQFKHIHLAMTAIYAS 550

IntA_6945_BBM67680.1 329 -----------NPHF---DHDFHDTRATYAANKLDLLLNI-PDLSTTQALKLLKDELGHKDLSVTMRYLT 383

IntB_6283_BCN27153.1 488 --------FELGSNY---EFTPHQLRRSFAYYLIGYEL---------CNFPQLKQQFSHVSMAMTRHYAK 537

XerD_Eco_IEU92_RS14660 237 ------------DSE---KLSPHVLRHAFATHLLNHG----------ADLRVVQMLLGHSDLSTTQIYTH 281

Int_SXT_AAL59748.1 333 ----------SVEME---HWSMHDLRRTMRTRMSAIT-----------TQEVAELMIGHSKKGLDAIYNQ 378

IntA_6283_BCN27155.1 388 ----------VDRSW---YYRIHDLRSTFATHWLWNESKE-RDVGYDYLMDELKELMGHSSTSITEKYIK 443

[Consensus_aa:](http://prodata.swmed.edu/promals3d/info/consensus.html) ..........ss..b....*h*s.**H**p*h***R**.o*h*t*h*.*hh*.............*h*s...*h*p.b*h*.**H**.p.s*hh*..**Y**.p

[Consensus_ss:](http://prodata.swmed.edu/promals3d/info/consensus_ss.html) hhhhhhhhhhhh hhhhhhhh hhhhhhhhh

Conservation:

IntA_JBBCAEG-19-0032 456 -------YKSRL----------QWKN-------------------------------------------- 464

IntB_JBBCAEG-19-0032 499 GSAFAVNFLNTE----------DAKNLIDSMEYERQKAQYIDYEANVINTTSRLWGGEGNRIQVARDKGQ 558

Int_lambda_NP_040609.1 345 -------DRGRE---------------------------------------------------------- 349

IntI1_R388_HXF36_RS00185 315 -------VLKVG---------------------------------------------------------- 319

IntB_6945_BBM67682.1 551 -------HSEVLTLLGIQN-PAKIKEKIQEQEIE-SHAEYLKDMI---EHPEEQSGGFMKFMSGEPMV-- 606

IntA_6945_BBM67680.1 384 -------HWEGN----------PTKNQV---------PELMMALL---EEEHII---------------- 408

IntB_6283_BCN27153.1 538 -------NASKF--QKIRQKKKTLANEIDEERID-QKARVYLSIYEKLANKEKIAGGKGKEFAKSITKTN 597

XerD_Eco_IEU92_RS14660 282 -------V--------------------ATERLR----QLHQQHH-------PRA--------------- 298

Int_SXT_AAL59748.1 379 -------YQYLD-----------------------EMRHAYDVWYQQLE---------------TIIEPT 403

IntA_6283_BCN27155.1 444 -------YMNKL----------DDQI---------------NMAKAKNL---KINGGWS----------- 467

[Consensus_aa:](http://prodata.swmed.edu/promals3d/info/consensus.html) ......................................................................

[Consensus_ss:](http://prodata.swmed.edu/promals3d/info/consensus_ss.html) h hhh hhhh hhhhhhhh h

Conservation:

IntA_JBBCAEG-19-0032 ----------------------------------------------------------------------

IntB_JBBCAEG-19-0032 559 PLIITT--DRAM-----TERKFLKGELAYKSGPIG-GCTNLEPCDRISFTSIFACI--DCEKSILDDDRS 618

Int_lambda_NP_040609.1 ----------------------------------------------------------------------

IntI1_R388_HXF36_RS00185 ----------------------------------------------------------------------

IntB_6945_BBM67682.1 607 ------------VGDARFNQLVEDTKGANKSTGFG-TCFSGELCSMGHLFEPSKCVGRDCENLNVNKVEA 663

IntA_6945_BBM67680.1 ----------------------------------------------------------------------

IntB_6283_BCN27153.1 598 RNLFTDKVDNDMLTLNFWKKQIRNKKRHLHAVAPGIYCTST-TCGLRTLINLIECV--DCKNDYI--VDA 662

XerD_Eco_IEU92_RS14660 ----------------------------------------------------------------------

Int_SXT_AAL59748.1 404 GFPFNWRFGQ------------------------------------------------------------ 413

IntA_6283_BCN27155.1 ----------------------------------------------------------------------

[Consensus_aa:](http://prodata.swmed.edu/promals3d/info/consensus.html) ......................................................................

[Consensus_ss:](http://prodata.swmed.edu/promals3d/info/consensus_ss.html)

Conservation:

IntA_JBBCAEG-19-0032 465 ------------------------------------SVQHEFETQLFRHVN---TSANA----GATV--- 488

IntB_JBBCAEG-19-0032 619 LKK---IKRGLNNLRREQAFYVAENAQYMQLESEIAAIYE---KLEKRGLR---KKMEALA--------- 670

Int_lambda_NP_040609.1 350 ----------------------------------WDKIEIK----------------------------- 356

IntI1_R388_HXF36_RS00185 320 ----------------------------------GAGVRSPLDALPPLTSER------------------ 337

IntB_6945_BBM67682.1 664 ENWVIRRERCIEKIEKMKEMGMFNRSSLATQLSDIRTAEK---VMADHNIQFEKYRVEAL---------- 720

IntA_6945_BBM67680.1 ----------------------------------------------------------------------

IntB_6283_BCN27153.1 663 VFAEAKRKEAEIHMLYDIENNELTPQTASESYIKVQAAER---IMSDLGIDYEPVVFPNEVRDLLIPFGV 729

XerD_Eco_IEU92_RS14660 ----------------------------------------------------------------------

Int_SXT_AAL59748.1 ----------------------------------------------------------------------

IntA_6283_BCN27155.1 ----------------------------------------------------------------------

[Consensus_aa:](http://prodata.swmed.edu/promals3d/info/consensus.html) ......................................................................

[Consensus_ss:](http://prodata.swmed.edu/promals3d/info/consensus_ss.html) hhhhhh h

Conservation:

IntA_JBBCAEG-19-0032 --

IntB_JBBCAEG-19-0032 --

Int_lambda_NP_040609.1 --

IntI1_R388_HXF36_RS00185 --

IntB_6945_BBM67682.1 --

IntA_6945_BBM67680.1 --

IntB_6283_BCN27153.1 730 FS 731

XerD_Eco_IEU92_RS14660 --

Int_SXT_AAL59748.1 --

IntA_6283_BCN27155.1 --

[Consensus_aa:](http://prodata.swmed.edu/promals3d/info/consensus.html) ..

[Consensus_ss:](http://prodata.swmed.edu/promals3d/info/consensus_ss.html)

**FIG S1.** Secondary structure alignment of IntA (JBP_33480) and IntB (JBP_33500) of JBBCAEG-19-0032 with known tyrosine recombinases. R-H-R-Y motif was highlighted by yellow background. The alignment was generated using PROMAL3D. e: sheet; h, helix.
