# Supplementary material for: Nationwide genome surveillance of carbapenem-resistant Pseudomonas aeruginosa in Japan
Source: Antimicrob Agents Chemother. 2024 Apr 2;68(5):e01669-23. doi: 10.1128/aac.01669-23 (PMC11064530; doi:10.1128/aac.01669-23)
Supplement: Fig. S2 — Secondary structure alignment of JBP_33490 product of JBBCAEG-19-0032 with known Tfp homologs. [file aac.01669-23-s0002.docx]

Conservation: 9 6 66

WP_013855472.1_VagAT68554 1 MAKSRLSVSRNQNKPITQLKAKD------NVTSLDLS---FELHQRGMAYSIKYNLLDWCHE-------- 53

BCN27154.1_6283 1 MAKSKIA---KQSKAVKHVRNNQ------NVVPLNLD---IPYKGTRETRVKLFDVSHLLYF-------- 50

WP_017821119.1_ValAT17749 1 MTE------------LTYLSGWR----------SDLC---IKLDV---RDNLLADFNRLLFN-------- 34

BCN27178.1_6945 1 MTENFDSDKPDNVRTYRSIPTDEMQSDNPLSKIHQWK---YIWVS-GNGGNRTCDFKGWLKY-------- 58

JBBCAEG-19-0032_JBP_33490 1 MI-SDLV---QRS--VVIIDAKDK-----NVEIIHPQLTVLMFDNKSGAGKRHLDLGALCYVRRGRGTPQ 59

WP_014386728.1_Vda04Ya311 1 MSRK------DNSKPVVLDDAFYEAPAA-TVEPLHLDELKLK------CGGATSHFIQLLYQ-------- 49

[Consensus_aa:](http://prodata.swmed.edu/promals3d/info/consensus.html) **M***h*cp......pps..*l*..*l*ps.p.......sp.*h*p*h*p...*h*b*h*.s...s.s.*hh*c*h*..*hh@*.........

[Consensus_ss:](http://prodata.swmed.edu/promals3d/info/consensus_ss.html) eee eee eee eee eeeehhhhhh

Conservation: 7 6 7

WP_013855472.1_VagAT68554 54 --QCDP----QK------PLVKPSRLNRMQKLRAWVNQERKNQSSEAYIRVKLASLKQYISFCDFNMLD- 110

BCN27154.1_6283 51 --GSNK----EN------KKI-GNRTVFIRSFCKKAHQYVSNGKSATSVTGCYESLRAYVAFCDALKVD- 106

WP_017821119.1_ValAT17749 35 --ELPS----SKELQQSNQFKFADREAFVFQLKDSFSEKIDEGVSHDSLYGIYVEASQYLRWCDKESEP- 97

BCN27178.1_6945 59 -----------------------STKEHVSLLISKFEQ---SGWAEASKVRNFQHIDNILKHNFTSQSQK 102

JBBCAEG-19-0032_JBP_33490 60 KMGCPV----DL------LSIDHSRIPVAQGLI----EHLRGSASIATATQVFQAISCFFDWIDDQPRR- 114

WP_014386728.1_Vda04Ya311 50 --GAPNYSQQGKKIKGVDYIPVAGREAFVRDAYRLLRTDFN-----RTKQNHFEQLRLYIRWMDREHRK- 111

[Consensus_aa:](http://prodata.swmed.edu/promals3d/info/consensus.html) ...*h*ss.....b......b....s**R**bs*hh*p.*h*.pb*h*ppc*h*p.t.t.so....*@*.p*l*p.*@l*p*@h***D**.p..p.

[Consensus_ss:](http://prodata.swmed.edu/promals3d/info/consensus_ss.html) hhhhhhhhhhhhhhhhh hhhhhhhhhhhhhhhhhhhhh

Conservation: 9 7 7 7

WP_013855472.1_VagAT68554 111 -----PF------SQGGYLSYVGNSGQLWRLVNAANEPKRYHFQYHDGEEAGLLEDTAFIQKTLLDILLT 169

BCN27154.1_6283 107 -----PF------SESGYLKYAGNDGELRHRIKMFTPSKRL-WEYNHGDELSKKESSASTVLSTLRTALE 164

WP_017821119.1_ValAT17749 98 -----AF------TQSSLEGYM---VHLQTRVMLGE----------------LKSSTYKNRRANMVTLFS 137

BCN27178.1_6945 103 RKSKVEF------SPESCVNYI---QAVWLSQRTTGVG-------LHGK--PIKAKTLGWLASNYNSILK 154

JBBCAEG-19-0032_JBP_33490 115 -----CFFDDVAAMQKGYGDYT---QHLLHRMNSSGV---------NGK--PIKKTTASQYQAGARKVLM 165

WP_014386728.1_Vda04Ya311 112 PINGDYF------APDLYNAFM---DYHQDKCNRGEQ----------------SLSTWSNAKRIVSFFLK 156

[Consensus_aa:](http://prodata.swmed.edu/promals3d/info/consensus.html) ......**F**......s.pt*h*.s**Y***h*.....*l*bpp*h*p.s.................*l*p.s**T***h*....s.*h*p.*h***L**p

[Consensus_ss:](http://prodata.swmed.edu/promals3d/info/consensus_ss.html) hhhhhhhh hhhhhhhhh hhhhhhhhhhhhhhhhh

Conservation: 7

WP_013855472.1_VagAT68554 170 VL-DFD----VSEWQATLKPFSIKNSESSTQPY-TSSEWYALVRRTQLFFFSLATQLIAFKEENPEAPPP 233

BCN27154.1_6283 165 WC-GLP----VSDWARHHRGFA--RENEPFKGY-SDDEETLLVTRLDTLFFTLAPQLIAAKENNT--LLP 224

WP_017821119.1_ValAT17749 138 RYLDLP----HYYFNNVVIMDN--SDRESYESY-TQSDLKQLLPFLRRLFNQTYSQFIKNPE-------- 192

BCN27178.1_6945 155 KF-GFG------DIPKATRNLDTASASLDSNNY-TKKELKRIARALLSDRKLLYKEYLDKT--------- 207

JBBCAEG-19-0032_JBP_33490 166 LTTGLS-EPEVKGIATHISQKDGQSRHINLALP-NADEQARTFAVLVNFIDEAHRILVQGGA-------- 225

WP_014386728.1_Vda04Ya311 157 AY-NRPIEAKQLILIK---GTK--KQANSHKGIDVVGELKPLVRRFIAAFAGFRRHFLEGT-------KP 213

[Consensus_aa:](http://prodata.swmed.edu/promals3d/info/consensus.html) .*h*.s*h*s.......*h*.p*hh*...s..ppp.s*h*ps*h*.s.s**E**b..*lh*..*h*..*hh*..*hh*pp*hl*............

[Consensus_ss:](http://prodata.swmed.edu/promals3d/info/consensus_ss.html) hh hhhhh hhhhhhhhhhhhhhhhhhhhhhhh

Conservation:

WP_013855472.1_VagAT68554 234 QCLEGI--------VVDRNNGRDITITLRGKD-------------------------------------- 257

BCN27154.1_6283 225 ETLPLIISLGLHEETLHIPTSLETRFASKNGTA------------------------------------- 257

WP_017821119.1_ValAT17749 193 ---AHI----------QAHKSTPTMIFEWQGRE------------------------------------- 212

BCN27178.1_6945 208 ------------------------------ISE------------------------------------- 210

JBBCAEG-19-0032_JBP_33490 226 ---FPL--------CLASPNGESVYLYSEQVDTKKAKAANFSLAPFLANSPTFPTWGEIKAHFGLEGGSA 284

WP_014386728.1_Vda04Ya311 214 SLHPLWSEELFNQ-QAE-KN--GWSVKKRNQVKGTFKRTV------------------------------ 249

[Consensus_aa:](http://prodata.swmed.edu/promals3d/info/consensus.html) .....*h*.............p..s*h*.*l*.p.p.sp.....................................

[Consensus_ss:](http://prodata.swmed.edu/promals3d/info/consensus_ss.html) h eee eeeee

Conservation: 6 7 9 7 6

WP_013855472.1_VagAT68554 258 -----------GSVSPF---------------NQCMAAAYTLFAYYTAFNDTVIKDVRHPI-KVITSKVE 300

BCN27154.1_6283 258 ----------VNAGAAF---------------NLTMGAAYHLMCFFTSQNDSNIRNIAHPI-HVHTEDR- 300

WP_017821119.1_ValAT17749 213 ----------YKLCGGI---------------TKMMCAATYLLAYYTYANTTDLFRLKQPN-NASIS--- 253

BCN27178.1_6945 211 ----------YKREIAF---------------NRLICNAVFLYVYYTAVGQAEALNTFVVEDSWSID--- 252

JBBCAEG-19-0032_JBP_33490 285 ATQKQRATYDYRRRIYLSNNDNLRSCLRQRIGSHAVAAGMLAFIAATSCNFSVAKNLEVD--LLEIV--- 349

WP_014386728.1_Vda04Ya311 250 ----------VGENAAR---------------NHFSRLAAMIAFCFTGQNTTPLLNLRFSD-IRFTA--- 290

[Consensus_aa:](http://prodata.swmed.edu/promals3d/info/consensus.html) ..........*h*....s*h*...............sp*hh*t*h***A***hhlhhh@***T**t.**N**pos*h*bs*l*.*h*s...*h*.*h*s...

[Consensus_ss:](http://prodata.swmed.edu/promals3d/info/consensus_ss.html) hhhhhh hhhhhhhhhhhhhhh hhhhhh eeee

Conservation: 9 9 6 76 7

WP_013855472.1_VagAT68554 301 GRTSKTAQVRAYKSRAS-KDVKALFAGSDENLHSEAIEKEAGFIVAEINKRDAVGKIDGITFIQTLELLS 369

BCN27154.1_6283 301 DKSLQVVKVSSYKPRSS-NEVDALLVGE----------------SFDVNKR------DGVKFIKLLERLS 347

WP_017821119.1_ValAT17749 254 -TGETWYTMPAFKRRAF-KTIQVEIGEH----------------ELEIPKY-------AMTFFDKLLNAS 298

BCN27178.1_6945 253 KAGGNRISVKGLKTRGY-KEESRTFA---------------------PRAV-------SKTFFEEHFELS 293

JBBCAEG-19-0032_JBP_33490 350 -PTSQGNRLSGTKGRARGKKVSPEFG-----------------------AR-------FAPVFKKYLDLR 388

WP_014386728.1_Vda04Ya311 291 -KSDGKVYFDMTKARANYLGFDTSLG---------------------FHKK-------TQAFFHQWLEVS 331

[Consensus_aa:](http://prodata.swmed.edu/promals3d/info/consensus.html) ..s.p.*h*p*h*pt*h***K**s**R**t..pp*h*ps.*h*t......................p.........s.s**F***h*cp*h*bp*l***S**

[Consensus_ss:](http://prodata.swmed.edu/promals3d/info/consensus_ss.html) eeeeeeee eeee ee hh hhhhhhhhhhhh

Conservation:

WP_013855472.1_VagAT68554 370 KSYSND-PFDTLIYFLDKEGE---KDKVNVSHALLKLS--QNLNLLSSRRGELTEHLIKTYTDIVENQNM 433

BCN27154.1_6283 348 KLYGNSEDGSELLFTLNNNSE--VSNAFNLRELNKALV--NKLHLLSPHRAGNLPWFK-ELFYTYRNQQS 412

WP_017821119.1_ValAT17749 299 RIISTN-EDATLLQTIASRKVQ-KLQTSRLQSFLKKWV--EKHFSFTDQMGRR----------------- 347

BCN27178.1_6945 294 KLNAVDLGLDKHYLFTRRNGN--QPTPMNLNTYIASLM--TRSVLLQ----------------------- 336

JBBCAEG-19-0032_JBP_33490 389 KWVLNGAESTLVFPFFSPQYGF-VSLTGHQINRTKILF--NKALPHT----------------------- 432

WP_014386728.1_Vda04Ya311 332 MVLQKNAGTEWVFPYFRKSGEIQGCVDAGQTTPQKSINKCTKILGLA----------------------- 378

[Consensus_aa:](http://prodata.swmed.edu/promals3d/info/consensus.html) **+***hh*.ss...s.*lh*.*hh*ppp.......s.pbpp..b.*l*...p**+***hh*.*h*s.......................

[Consensus_ss:](http://prodata.swmed.edu/promals3d/info/consensus_ss.html) hhh eeeeee h hhhhhhhhhhhh hhh

Conservation:

WP_013855472.1_VagAT68554 434 PIFNWSQREDGARTMNKQVIYLNKRTATKRSTPIAYAVFSCMADVSLRNALIPLHYAEKDANGEITVSFK 503

BCN27154.1_6283 413 ITLKKVTNHLGRTVVHKEVQEVSKTKAGQGATNSAYCILSCYTDLPYKGVLLPLTYSEIDSDGNVTVSFK 482

WP_017821119.1_ValAT17749 ----------------------------------------------------------------------

BCN27178.1_6945 337 -TMKA----------------------------------------------------------------- 340

JBBCAEG-19-0032_JBP_33490 ----------------------------------------------------------------------

WP_014386728.1_Vda04Ya311 ----------------------------------------------------------------------

[Consensus_aa:](http://prodata.swmed.edu/promals3d/info/consensus.html) ......................................................................

[Consensus_ss:](http://prodata.swmed.edu/promals3d/info/consensus_ss.html) eee

Conservation:

WP_013855472.1_VagAT68554 504 YVDGSEGEFTVTAKHLPFLQLVERHA---ATRNPLPKERSLGGIGRRSNATKPPFLLPLGTKSMTYQWQE 570

BCN27154.1_6283 483 YRSGETGYFKAPACDLTLIKDIERYAKEKANKQAKKYKRLLLTRSS---------------NNIPYDW-E 536

WP_017821119.1_ValAT17749 ----------------------------------------------------------------------

BCN27178.1_6945 341 ----ANPDF------------------------------------------------------------- 345

JBBCAEG-19-0032_JBP_33490 ----------------------------------------------------------------------

WP_014386728.1_Vda04Ya311 ----------------------------------------------------------------------

[Consensus_aa:](http://prodata.swmed.edu/promals3d/info/consensus.html) ......................................................................

[Consensus_ss:](http://prodata.swmed.edu/promals3d/info/consensus_ss.html)

Conservation: 7 6 7 9

WP_013855472.1_VagAT68554 571 GEVPISASMLSYCGIGYGDYFLNINSRRIRVTHSDLEYKPE-ERGMTAQKILQHSIDTAD--KKYRNGHP 637

BCN27154.1_6283 537 GISPISANLMKRWSVEPNHYYLSLQSSRWREMSSNQAYAEG-G-VQAVQSLLQNKRDTIE--RSYINGLP 602

WP_017821119.1_ValAT17749 348 ---------------------LRPSISRFRETGCQLTSYHQ-G-EMVNNLMLNNTPNTRK--RHYSEGNK 392

BCN27178.1_6945 346 ----------------------SLTCERLKSSIKQYAEEKL-G-RQGAMESNRNTSESTWNNSDYSKNSK 391

JBBCAEG-19-0032_JBP_33490 433 ---------------------SWVTPTQWRKGVSYQYVKLSGGDLALTAEKLGNTEATLR--QSYSRPAL 479

WP_014386728.1_Vda04Ya311 379 ----------------------HVTPSKLRQTKIDTLMKVT-EDIWLVSMSANNSIEVVA--INYSDGNE 423

[Consensus_aa:](http://prodata.swmed.edu/promals3d/info/consensus.html) ......................p*l*ssp**+***h***R**ps..pb*h*.b......b*hh*.b.*h*p**N**o.po*h*...pp**Y**pps..

[Consensus_ss:](http://prodata.swmed.edu/promals3d/info/consensus_ss.html) hhhhhhhhhhhhhhhh hhhhhhhh hhhhh hhh h

Conservation: 6 9 9

WP_013855472.1_VagAT68554 638 VQNNKQVSQGLMALSRIAEG--ETRNEAIESVKAELKIPVLEYEVWKKRNQ------PTNPNG-ISCDG- 697

BCN27154.1_6283 603 SLNKVILSQGIEVIENLFDN---DLEKAKDAVAKRRGIPMLTYEESEKKRK-------TNPNG-IVCDG- 660

WP_017821119.1_ValAT17749 393 LANNGMMQDTMSIREEQVKS-----RVNTKQAQKNLSIEVLVIEEENKINL---PNLSRTSNG-GSCAA- 452

BCN27178.1_6945 392 ALAHRELAFGTATLFALGSNPTGGVTAAIATTKAQHC-EVLSSEEVDALRASSSEPVDAIANG-GVCKG- 458

JBBCAEG-19-0032_JBP_33490 480 EDFAAEMTVFFELMHKAA-------------IDRTRSVEHIPVRIVDEIRL-------DAVTGIGSCEKA 529

WP_014386728.1_Vda04Ya311 424 SDHRTSLAANNEALYDFSKNG-TDPHEAANKSKFNHANVLNGYDYKRLRKE-ETENDTQTPLG-VRCKD- 489

[Consensus_aa:](http://prodata.swmed.edu/promals3d/info/consensus.html) ...p.b*h*s.s*h*.*hh*.p*h*sps.....p.**A***h*.p*h*p.p.t..*hl*s*h***-**b.cb.pb.......psss**G**..s**C**cs.

[Consensus_ss:](http://prodata.swmed.edu/promals3d/info/consensus_ss.html) hhhhhhhhhhhhhhhhhh hhhhhhhhhhhh hhhhhhh

Conservation: 9 7 9 9 9 69 779 9

WP_013855472.1_VagAT68554 698 --KIDLNSEKDWHYAARKFAEEKGIIKEGD--DITCYQYDLCPFCKSAKLVDDPYAIYKLLSFLDVFSEA 763

BCN27154.1_6283 661 --KQAM--------IDGKNTQRETNYALDA--DLPCAEFDMCHKCQSAKALDEVDAIYKLISYIDVLKEG 718

WP_017821119.1_ValAT17749 453 --PFGEKSQ-----KYTKKAQTQGLAKEGE--RLACADLLGCFGCPDQVIVQSVSDIWCLLSFKSCVEES 513

BCN27178.1_6945 459 --EDVPQKR-----EFQKSNLETGLLDDDDVKNTACGYVIKCFMCRNFAVVDEVHDIWRLLSFEFRLNEA 521

JBBCAEG-19-0032_JBP_33490 530 AETAPRR--------------AQGFTAQAP--APACGDPETCLFCEFYAVHADEQDIRRLLSLRYLIE-A 582

WP_014386728.1_Vda04Ya311 490 --SSQGVAV-----RIKKNLENMGVKQPEK--EQRCTDFLGCFECHYHRLVSEVEDIWLMLSFNDTLQEM 550

[Consensus_aa:](http://prodata.swmed.edu/promals3d/info/consensus.html) ..p.s..........*h*p**K**p.bpp**G***hh*..s...p.s**C**sp*h*..**C***h*b**C**p.*h*.*ll*s**-**sps**I***@*.**LLS***@*..*hl*p**E**t

[Consensus_ss:](http://prodata.swmed.edu/promals3d/info/consensus_ss.html) hhhh hhhhhhhh hhhhhh eeee hhhhhhhhhhhhhhhhh

Conservation: 9 7 6 7

WP_013855472.1_VagAT68554 764 IDRY------PERASVIQSKIERFQEHLDDLP---FETIEKAEDLLEEKGRYPLFHSLS-SVTQFL---- 819

BCN27154.1_6283 719 LDMF------PDSKGDALEKIKAFECTLDEAS---NDVFDEAMKKFNTQGRHPRVSIDH-AILSM----- 773

WP_017821119.1_ValAT17749 514 LFLHLDVSHYKQNFENIILFINQ--KILPNIN---KQILRKAETKLDDDGLHPAWDDSE-SVLSLIPKSP 577

BCN27178.1_6945 522 VAAHKSLDHFIKNFSEVKSAIRDIKKRFK------KRNLKAAEKYLERQGCHPLWDED--SIQDIFKG-- 581

JBBCAEG-19-0032_JBP_33490 583 IKHKQPIDHWQTKFSPTLHRIDEVLSAIQDADGRIKPTVTRVRDEVASGAFDAFWSIHFDTLVTVGAVT- 651

WP_014386728.1_Vda04Ya311 551 KAYPAINSLPTDKFYKLCNTIESILKDFKEVA---PDNYTRAQEMH-SEAPHPLYSDGY-SLMDLLETF- 614

[Consensus_aa:](http://prodata.swmed.edu/promals3d/info/consensus.html) *l*.*h*........pp*h*..*h*bp.**I**cp*h*bp.*h*pp*h*s....ps*h*pc**A**pcb*h*.ppt.c**P***h@*s....o*lh*s*h*.....

[Consensus_ss:](http://prodata.swmed.edu/promals3d/info/consensus_ss.html) hh hhhhhhhhhhhhhhhhhhhhh hhhhhhhhhhhhh hh hhhhh

Conservation:

WP_013855472.1_VagAT68554 ----

BCN27154.1_6283 ----

WP_017821119.1_ValAT17749 578 AEVR 581

BCN27178.1_6945 ----

JBBCAEG-19-0032_JBP_33490 ----

WP_014386728.1_Vda04Ya311 ----

[Consensus_aa:](http://prodata.swmed.edu/promals3d/info/consensus.html) ....

[Consensus_ss:](http://prodata.swmed.edu/promals3d/info/consensus_ss.html)

**FIG S2.** Secondary structure alignment of JBP_33490 product of JBBCAEG-19-0032 with known Tfp homologs. The alignment was generated using PROMAL3D. e: sheet; h, helix.
