# Supplementary material for: Nationwide genome surveillance of carbapenem-resistant Pseudomonas aeruginosa in Japan
Source: Antimicrob Agents Chemother. 2024 Apr 2;68(5):e01669-23. doi: 10.1128/aac.01669-23 (PMC11064530; doi:10.1128/aac.01669-23)
Supplement: Fig. S3 — Secondary structure alignment of JBP_33510 of JBBCAEG-19-0032 with known Srap homologs. [file aac.01669-23-s0003.docx]

Conservation: 9 7 97 77 6 7 797 7

WP_013855470.1_VagAT68554 1 MTDRTE--------LSSTEKQLREALKRLIDKQPTHR---ELKRKLSANKL-KIVVANVEKEAGLSNGAA 58

WP_014386726.1_Vda04Ya311 1 MNQETLRALVQEEKTLAAEEKLHKALARLQNGKPLRT----------KAKG-RLTLNKINNEAGLGRSYI 59

BCN27180.1_6945 1 MA--------------TVATKLENAFKALLKEGK------------------KISPYAVERRAGVSNGSL 38

WP_017821121.1_ValAT17749 1 MSK-----------SENTLLKLEAALERIKQGKPKRI----------PTHR-KLSVRAVEEEANLGNGSG 48

BCN27152.1_6283 1 MS-------------QTTKKKLIDALERLLSGDVAKLTSRELRNKA-RKGKLKINNSNVEKEAGLSVGAL 56

JBBCAEG-19-0032_ JBP_33510 1 MS---------------ALENYIAALQRLIAGKPQNV----------PKGS-AINKDTVALEAGRKRGSI 44

[Consensus_aa:](http://prodata.swmed.edu/promals3d/info/consensus.html) **M**s..............s*h*bpp**L**..**AL**p**RL**bp.ps.p................**+***l*s..s**V**pp**EAG***l*tp**G**t*h*

[Consensus_ss:](http://prodata.swmed.edu/promals3d/info/consensus_ss.html) hhhhhhhhhhhhhhh eeehhhhhhhh hh

Conservation: 6 776

WP_013855470.1_VagAT68554 59 KRY----PETKKMIEGAEAERIHGSSDVSGEVVRAHPIYIKARE----DLEK---AREEVKKLKAELEQK 117

WP_014386726.1_Vda04Ya311 60 HKF----KEFVDNVANPAIEAYNETLDTP----KPSEIDKAS-TEDMSEIDRLKDELRKQIELKEAY--- 117

BCN27180.1_6945 39 KNH----IVLLEKVLAEKEKYATDPTNVG--VVKARANKAKAKV----SKDKYQEVLDKNEKLRAEN--- 95

WP_017821121.1_ValAT17749 49 YYY----PDFVEKVKQTKGEIATGKGGIV----QPEILTVRTKLK---EQKR------IKDNYKAKYE-- 99

BCN27152.1_6283 57 RRH----NDVILMIKNKSLEVQVAQDETS----DSPIEILLKEIK---SIRS---EKIREKNLKEEYY-- 110

JBBCAEG-19-0032_ JBP_33510 45 KKSRAGNAELIAAIEAAAAAQQEKSGPTA-------AQDATKQKA---LKRA---AQAQLGSLKEDY--- 98

[Consensus_aa:](http://prodata.swmed.edu/promals3d/info/consensus.html) **+**p*@*.....**-***hl*..*l*bs...c..p.pss*h*s....csp.b.*h*..p.....pbcp....b.p..p**LK**.c*h*...

[Consensus_ss:](http://prodata.swmed.edu/promals3d/info/consensus_ss.html) hhh hhhhhhhhhhhhhhhh hhhhhhhhhhhh hhhh hhhhhhhhhhhhh

Conservation: 66 6

WP_013855470.1_VagAT68554 118 DGRLAHYKELLKSQAARMHQMNVAMWNDIPEEKKHVEVIIDVQDMG---------SQD---NILAFKKRE 175

WP_014386726.1_Vda04Ya311 118 -------RTERDEALATNDELEVLN----------KSLMFRVYELQ-------QELTD---SVAEYEGYK 160

BCN27180.1_6945 96 ------------------EQFKGEL----------KVMADKVAQMTWELHRYKTKTRKDSTNVHNLKA-- 135

WP_017821121.1_ValAT17749 100 -----AEREKLALFAAAQHHLNDKL----------VQALARIDDLEY--ENA--ELRE---ELAKLKRSK 147

BCN27152.1_6283 111 -----GEAQNHQDALAAQAATHVKV----------VQE---LMDMLHESERE--KAMD---KVVNARPDN 157

JBBCAEG-19-0032_ JBP_33510 99 -----ELALTRIASLVHENHTLKMQ----------IKE---LVE-----EKE--WSKH---KVVKMNEAK 140

[Consensus_aa:](http://prodata.swmed.edu/promals3d/info/consensus.html) ..............*hh*..pp*h*psb............p....*l*.**-***h*.........c.pc...p*lh*p*h*c..c

[Consensus_ss:](http://prodata.swmed.edu/promals3d/info/consensus_ss.html) hhhhhhhhhhhhhhhhhhhh hhhhhhhhhhhh hhh hhhh hhhhhh

Conservation:

WP_013855470.1_VagAT68554 176 KLN----- 178

WP_014386726.1_Vda04Ya311 161 K------- 161

BCN27180.1_6945 --------

WP_017821121.1_ValAT17749 148 VVPIK--- 152

BCN27152.1_6283 158 IVEGHFRK 165

JBBCAEG-19-0032_ JBP_33510 141 RP------ 142

[Consensus_aa:](http://prodata.swmed.edu/promals3d/info/consensus.html) ........

[Consensus_ss:](http://prodata.swmed.edu/promals3d/info/consensus_ss.html)

**FIG S3.** Secondary structure alignment of JBP_33510 of JBBCAEG-19-0032 with known Srap homologs. The alignment was generated using PROMAL3D. e: sheet; h, helix.
